# Nonsense-Mediated mRNA Decay Controls the Changes in Yeast Ribosomal Protein Pre-mRNAs Levels upon Osmotic Stress

**DOI:** 10.1371/journal.pone.0061240

**Published:** 2013-04-19

**Authors:** Elena Garre, Lorena Romero-Santacreu, Manuela Barneo-Muñoz, Ana Miguel, José E. Pérez-Ortín, Paula Alepuz

**Affiliations:** Departamento de Bioquímica y Biología Molecular, Facultad de Ciencias Biológicas Universitat de València, Valencia, Spain; CNRS UMR7275, France

## Abstract

The expression of ribosomal protein (RP) genes requires a substantial part of cellular transcription, processing and translation resources. Thus, the RP expression must be tightly regulated in response to conditions that compromise cell survival. In *Saccharomyces cerevisiae* cells, regulation of the RP gene expression at the transcriptional, mature mRNA stability and translational levels during the response to osmotic stress has been reported. Reprogramming global protein synthesis upon osmotic shock includes the movement of ribosomes from RP transcripts to stress-induced mRNAs. Using tiling arrays, we show that osmotic stress yields a drop in the levels of RP pre-mRNAs in *S. cerevisiae* cells. An analysis of the tiling array data, together with transcription rates data, shows a poor correlation, indicating that the drop in the RP pre-mRNA levels is not merely a result of the lowered RP transcription rates. A kinetic study using quantitative RT-PCR confirmed the decrease in the levels of several RP-unspliced transcripts during the first 15 minutes of osmotic stress, which seems independent of MAP kinase Hog1. Moreover, we found that the mutations in the components of the nonsense-mediated mRNA decay (NMD), Upf1, Upf2, Upf3 or in exonuclease Xrn1, eliminate the osmotic stress-induced drop in RP pre-mRNAs. Altogether, our results indicate that the degradation of yeast RP unspliced transcripts by NMD increases during osmotic stress, and suggest that this might be another mechanism to control RP synthesis during the stress response.

## Introduction

Ribosome biosynthesis is a major consumer of cell resources. In each situation, cells must decide whether to dedicate a significant part of cellular energy to the production of active ribosomes or to slow down or inhibit ribosome synthesis. Under optimal conditions, unicellular *Saccharomyces cerevisiae* produces around 2000 ribosomes per cell and minute [1]. Yeast ribosomes are large ribonucleoprotein particles containing 4 ribosomal RNAs and 78 different proteins. Ribosomal proteins (RP) are encoded by 137 different genes scattered around the genome, 59 of which are duplicated. The majority (73%) of yeast RP genes contains introns, although only about 280 genes (5% of all yeast genes) are intron-containing genes in *S. cerevisiae*, and alternative splicing has been reported for very few cases [2], [3], [4]. The production of yeast ribosomes requires the dedication of a considerable part of RNA polymerase II activity to RP genes’ transcription, estimated in around 10% of total polymerase II transcription [5]. In addition, 90% of yeast mRNA splicing is devoted to the production of mature RP mRNAs. Moreover, one third of total yeast mRNAs corresponds to RP, implying that around 34% of total ribosomes work in the translation of RP transcripts. Therefore, the energy economy of yeast cells demands a coordinated and fine-tuned gene expression of RP genes, which is executed at the transcriptional and post-transcriptional levels [5], [6], [7].

Accumulating data evidence that the presence of introns in many RP genes is one of the factors that contributes to the regulation and coordination of ribosomal protein expression. Firstly, ribosomal intro-containing genes produce more RNA and more protein than ribosomal genes without introns [8], [9]. Secondly, experiments with deletions of RP introns have an impact on the gene expression of the host gene with both positive and negative effects. Moreover, introns are preferentially conserved (80%) in duplicated RP genes and play a role in the intergenic regulation of duplicated genes [4]. Finally, the biological impact of deleting yeast RP gene introns has been revealed through cell fitness and drug response experiments. Intron deletions did not display growth defects under normal growth conditions. However, in the presence of some drugs, such as treatments with NaCl, several yeast strains carrying RP intron deletions showed changes in cell fitness. These results suggest that the regulation of RP gene expression by introns is relevant under stress conditions [4], [10].

The steady state of intron-containing pre-mRNAs results from the balance among the transcription rate, the splicing rate and the rate of nuclear and cytoplasmic degradation [5]. Recently, splicing microarrays were employed to reveal that different stresses cause distinct patterns in RP pre-mRNA processing. RP pre-mRNAs accumulate under amino acid starvation conditions, which correlates with splicing inhibition. Glucose depletion or heat-shock decreased both the pre- and mature RP mRNA through a mechanism dependent on both nutrient-sensing casein kinase 2 (CK2) and the exosome nuclear mRNA degradation pathway. Under hyperosmotic stress, RP pre-mRNA decreased and mature species slightly reduced, and both effects seemed independent of CK2 and exosome [11], [12].

Nonsense-mediated mRNA decay (NMD), together with other mRNA degradation pathways, can eliminate unspliced pre-mRNAs. NMD, which is conserved among eukaryotes, directs the cytoplasmic mRNAs carrying intronic premature translation-termination codons (PTCs) to degradation through the action of three core universal factors namely Upf1, Upf2 and Upf3 (reviewed in [13], [14], [15], [16]). Thus, NMD acts as an RNA surveillance pathway for unspliced pre-mRNAs that are able to rich the cytoplasm and that frequently include PTCs. On those transcripts, a first translating ribosome stops when encountering a PTC, and Upf factors connect the premature translation termination event to mRNA degradation. In yeast, the mRNAs targeted by Upf proteins generally follow decapping and 5′ to 3′ mRNA degradation by exonuclease Xrn1. Apart from the elimination of aberrant transcripts, it has been proposed that mammalian and yeast cells routinely utilise NMD to achieve proper gene expression levels [17], [18], [19], [20], [21], [22] and that NMD might also be involved in preventing the accumulation of unspliced RP transcripts under physiological conditions that cause splicing inhibition [23]. The impact of NMD on the regulation of mammalian and yeast RP gene expression has started to be underscored [23], [24]. Recent results show that many yeast RP pre-mRNAs (31% of RP genes) accumulate through the inactivation of Upf1 and Xrn1. Targeting to NMD depends on the intronic features of RP pre-mRNAs, which also determine splicing efficiency. In mammalian cells, it has been estimated that 10% of “normal”, non-mutated mRNAs are regulated by NMD [17]. Some of these “normal” targets are stress-response genes degraded by NMD under non-stress conditions and induced by hypoxia by NMD repression [25]. Regulation of NMD by cellular stress can also have important implications for cancer since NMD targets several mutated tumor suppressor gene transcripts [26].

In *S. cerevisiae*, hyperosmotic shock regulates RP production and translation. Osmotic stress is detected by membrane sensors and signaling is transduced by the HOG MAP kinase pathway [27], [28]. After a few minutes of osmotic stress treatment, global changes in transcription, mRNA stability and translation are detected in yeast cells. The transient inhibition of translation correlates with the down-regulation of RP genes, which is executed through rapid transcriptional repression, mature mRNA destabilization and lower translation rates of the RP transcripts [29], [30], [31], [32], [33]. Under mild osmotic stress, cells resume growth after a short time (within minutes), global translation levels recover and the biosynthesis of ribosomes restarts.

In order to investigate the regulation of *S. cerevisiae* intron-containing genes under osmotic stress, we used yeast tiling arrays and discovered that the ratios between RP pre-mRNA and total RP mRNA levels lower upon stress. Our results show that stability of RP pre-mRNAs diminishes under osmotic stress and that the Upf and Xrn1 decay factors are involved in this regulation. Thus, osmotic stress modulates RP pre-mRNA levels via the NMD pathway. Our data highlight the importance of mRNA stability control as a mechanism for the fine-tuning regulation of gene expression in response to stress.

## Materials and Methods

### Yeast Strains and Growth Conditions

The wild-type W303-1A (MATa *ade2-1 can1-100 his3-11, 15 leu2-3, 112 trp1-1 ura3-1*) strain was used in the tiling array and genomic run-on experiments. Experiments with deletions *hog1Δ::KanMX4* [34] and *cbc1Δ::KanMX4* (obtained in this work) were done in W303-1A genetic background. Strains BY4741 (MATa *his3Δ1 leu2Δ0 met15Δ0 ura3Δ0*), as a wild-type strain, and mutants *hog1*, *cbc1*, *rrp6*, *xrn1*, *upf1*, *upf2* and *upf3* (obtained from Euroscarf) were used in the remaining experiments. Strains were grown in cultures with YPD media (1% yeast extract, 2% peptone, 2% glucose) at 30°C to the mid-log phase (OD600 0.5). Osmotic stress conditions were created by adding NaCl at 0.4 M and the incubation times are indicated in each case.

### Total RNA Extraction

To analyse cellular mRNA levels, exponentially growing cultures were incubated under the conditions and at the times indicated, and 20-ml cells culture were collected by centrifugation and frozen at −20°C. Total RNA was extracted from cell pellets using a FastPrep device (FastPrep 120, Bio101) in 0.5 ml of LETS buffer (0.1 M LiCl, 0.01 M EDTA, pH 8.0, 0.01 M Tris-HCl, pH 7.4, and 0.2% SDS [wt/vol]), 0.5 ml of phenol (pH 4.5):chloroform:isoamylic alcohol (25/24/1), and 0.3 ml of glass beads. Supernatants were extracted with phenol:chloroform:isoamylic alcohol (25/24/1) and chloroform:isoamylic alcohol (24/1). RNA was precipitated twice; first, by adding two volumes of 96% ethanol and 0.1 volumes of 5 M LiCl and then by incubating overnight at −20°C. After dissolving the RNA in RNase-free water, it was precipitated again by adding two volumes of 96% ethanol and 0.1 volumes of 3 M sodium acetate, and by incubating at −20°C for 3 h.

### Tiling Array

For the tiling array experiments, total RNA was extracted from exponentially YPD growing W303-1A cells before and after treatment for 15 minutes with 0.4 M NaCl. The hybridization of tiling array Affymetrix (*GeneChip*® *S. cerevisiae Tiling 1.0R Array*) with cDNA produced from total RNA, was carried out by following the Affymetrix protocol and using the *GeneChip*® *Hybridization, Wash and Stain Kit* (P/N 900720). The final data were obtained from the results of 3 independent biological experiments.

High-density tiling arrays images were normalized and analyzed using the Affymetrix Tiling Analysis Software, and were then visualized by the Integrated Genome Browser (Affymetrix).

### Genomic Run-on (GRO)

GRO experiments were conducted to obtain genome wide transcription rates (TR). GRO assays with the yeast cells grown until the exponential phase and then treated with 0.4 M NaCl at the indicated times were carried out as described in [30]. Each experiment was repeated 3 times. GRO hybridizations were normalised as described in [35]. Changes in the TR for RP genes were evaluated by K-means clustering using the Euclidean distance of the normalized averaged value. For the cluster analysis of the results, we used the MeV Multiple Experiment Viewer (http://www.tm4.org/mev/).

### Quantitative RT-PCR

Total cellular RNA was treated for 15 minutes at 25°C with DNase I RNase-free (Roche) according to the manufacture’s protocol and prior to use for cDNA synthesis. Then cDNA was synthetized in 20 µL reactions containing 25 ng/µL of DNase I treated RNA, 5 µM of Oligo d(T) (Thermo Scientific), 10 units/µL of SuperScript II Reverse Transcriptase (Invitrogen), 1×First Strand Buffer, 10 mM DTT, and 0.8 mM dNTPs. Afterwards, RNA and primers were denatured for 10 minutes at 65°C, First Strand Buffer and DTT were added and incubated for 2 minutes at 42°C. Finally, the remaining reagents were added, and the reaction was incubated at 42°C for 50 minutes and at 70°C for 15 minutes. Quantitative real-time PCR was then performed in a DNA Engine Peltier Thermal Cycler (Bio-Rad) using the SYBR® Premix Ex Taq™ kit (Takara) for fluorescent labelling. For this purpose, 2.5 µL cDNA were added to each reaction in a final volume of 10 µL. Real-time PCR reactions using 0.2 µM of the corresponding oligonucleotides were performed under the following conditions: 95°C for 10 seconds, followed by 40 cycles of 5 seconds at 95°C and 20 seconds at 52°C, and a final step at 50°C for 1 minute. At the end of the amplification cycles, a melting curve analysis was conducted to verify the specificity of the reaction. For each analysed primers pair, a negative control was included and a standard curve was made with serial dilutions of the untreated wild-type cells cDNA sample (2×10^−1^, 1×10^−1^, 2×10^−2^, 1×10^−2^, 2×10^−3^ and 1×10^−3^). To examine both the pre- and mature mRNA, specific primers to intron or exon regions were designed. The primers set used in this study is provided in Table S1. A primers pair for *ACT1* exon2 was used as a reference gene. Data and errors bars represent the average and standard deviation of 3 independent biological samples.

### PM Index (PMi) Calculation

The PMi is the ratio of the intron signal after a 15-minute incubation in the presence of 0.4 M NaCl (stressed cells) normalized by the intron signal in non-stressed cells, in relation to the exon signal in the stressed cells normalized by the exon signal in non-stressed cells. Therefore, the PMi from the tiling arrays data was calculated as: log_2_ [(it_15_/it_0_)/(et_15_/et_0_)], where it_0_ and it_15_ are the experimental values of intron intensity and et_0_ and et_15_ are the experimental values of exon intensity at time 0 and after 15 minutes of osmotic stress, respectively. In the quantitative RT-PCR experiments, the PMi was calculated as: log_2_ [(E^−ΔΔCt^)_intron_/(E^−ΔΔCt^)_exon_] using the “delta delta Ct” mathematical model described in [36], where E is the real-time PCR efficiency and ΔΔCt = (Ct_15sample_−Ct_15reference_)−(Ct_0sample_−Ct_0reference_), Ct_0sample_ and Ct_15sample_ are the experimental values of each primers pair and Ct_0reference_ and Ct_15reference_ are the experimental values of reference gene *ACT1*.

### Accession Numbers

The gene expression omnibus (GEO) accession number for the GRO experiments data is GSE13100 and is GSE43236 for the tiling array data.

## Results

### The Tiling Microarray Analysis of *S. cerevisiae* Response to Hyperosmotic Stress Shows a Decrease in RP Pre-mRNA Signals, which is Hog1-independent

**Figure 1:**
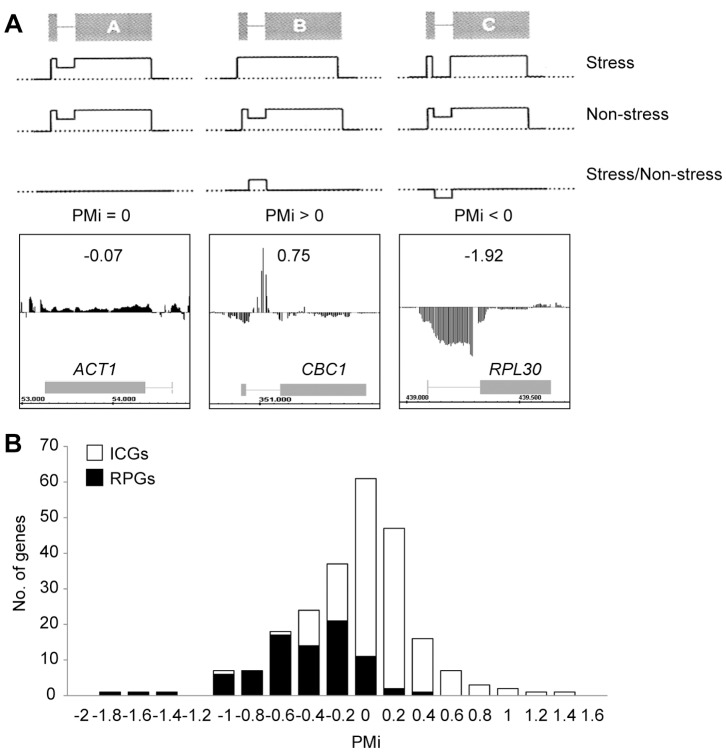
Tiling array analysis of exon and intron signals after osmotic stress in relation to non-stress. (A) Schema of the PM index (PMi) calculation using the intron intensity signal ratio after a 15-minute incubation in the presence of 0.4 M NaCl (stressed cells) in relation to non-stressed cells. The mRNAs without changes, with increases or with a decrease, in the intron signal in relation to the exon signal between the stress and non-stress conditions take values close to zero, or positive or negative PMi values, respectively. All these situations are illustrated with a real example (bottom panels). (B) Global distribution of ICGs (intron-containing genes) according to their PMi (see Material and Methods). The black boxes in the histogram represent the distribution of intron-containing ribosomal protein (RP) genes.

To investigate the putative changes in RNA splicing and/or the stability of yeast intron-containing genes (ICGs) upon hyperosmotic stress, we analyzed the signals obtained by the hybridization of the *S. cerevisiae* tiling arrays with the total RNA samples obtained from a wild-type strain before and after 15 min of treatment with 0.4 M NaCl. In order to compare the changes in the pre-mRNA levels in relation to total mRNA during osmotic stress, we calculated the PM index (PMi) as the ratio of the intron signal in the stressed cells normalized by the intron signal in non-stressed cells, and in relation to the exon signal in the stressed cells normalized by the exon signal in non-stressed cells (schematically shown in Figure 1A, see the Materials and Methods section for a mathematical definition). The genomic data allowed the calculation of the PMi for 234 ICGs (Table S2). For most of them (75%), the ratios of intron signals and exon signals during stress were between −0.5 and +0.5 (Fig. 1B). However, 20% of ICGs (47 genes) showed a PMi<−0.5, indicating that the intron/exon ratio significantly lowered during osmotic stress for these genes. This group was found to be enriched in the Gene Ontology category “cytosolic ribosome” (p-value of 1.229×10^−20^). Moreover, most of the RP genes containing introns gave a negative PMi (80 of 83) (Fig. 1B). The RP gene PMi average was −0.56, while the PMi average was +0.02 for the non-ribosomal ICG.

**Figure 2:**
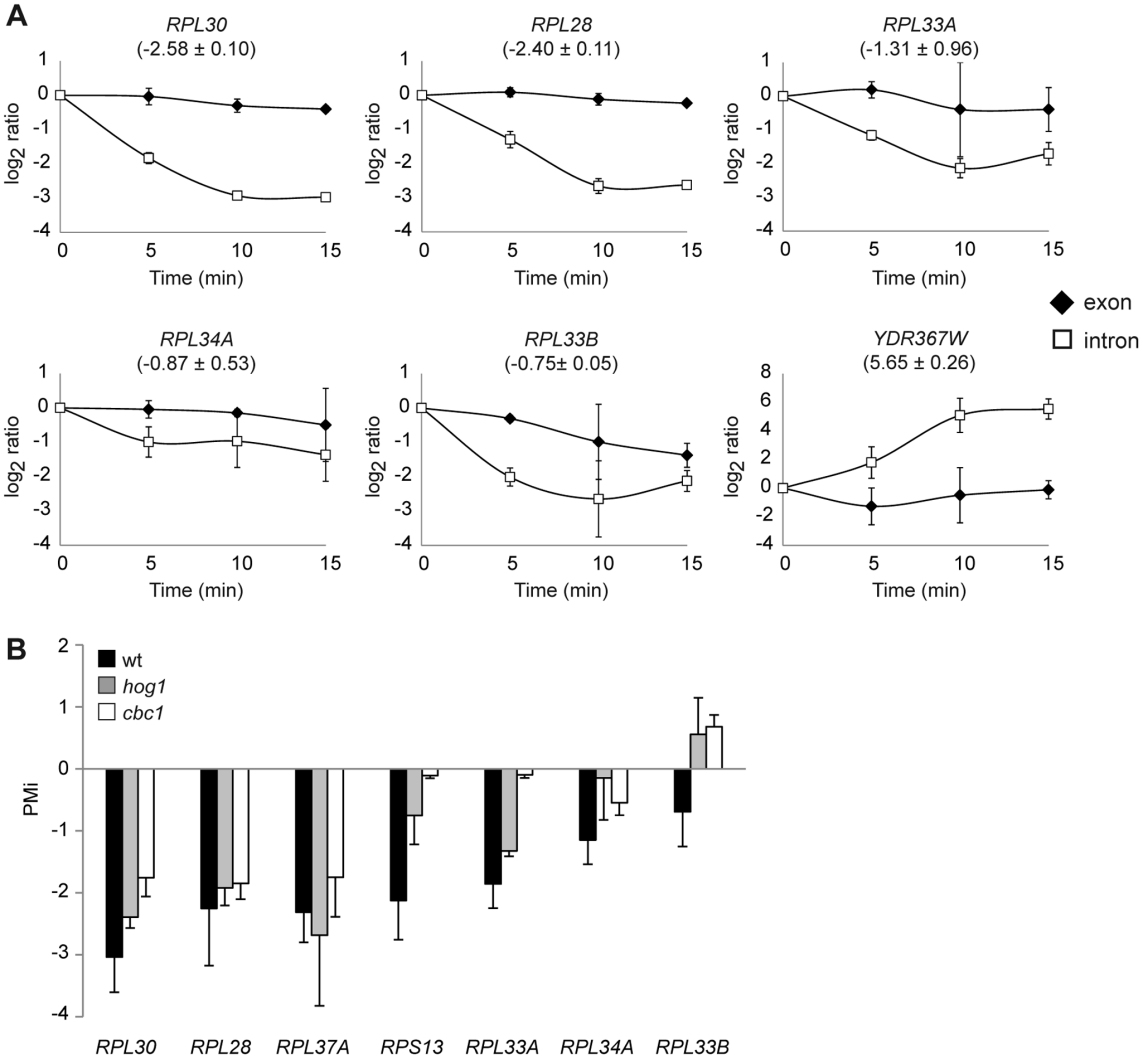
Quantitative RT-PCR analysis of PMi for ribosomal protein genes in wild-type and *hog1* or *cbc1* mutants. (A) A decrease in ribosomal protein (RP) pre-mRNAs in response to osmotic stress. The behaviour of transcripts *RPL30*, *RPL28*, *RPL33A*, *RPL33B*, *RPL34A* and *YDR367W* across a time course lasting 5, 10 and 15 minutes after treatment with 0.4 M NaCl was examined by quantitative RT-PCR using specific primers to intron regions and exon regions. The PMi value of the comparison made between stressed (0.4 M NaCl, 15 min) and non-stressed cells per gene, obtained by quantitative RT-PCR, is found in parentheses. Data and error bars represent the average and standard deviation of 3 independent experiments. (B) Representation of PMi for the wild-type, *hog1* mutant and *cbc1* mutant cells treated with 0.4 M NaCl for 15 minutes in relation to untreated cells. The intron and exon levels of the transcripts *RPL30*, *RPL28, RPL37A*, *RPS13*, *RPL33A*, *RPL34A* and *RPL33B* were examined by quantitative RT-PCR using specific primers to those regions. Data and errors bars represent the average and standard deviation of 3 independent experiments.

To validate the observed response to hyperosmotic stress, we calculated PMi values by quantitative RT-PCR experiments using both intron- and exon-specific primers of genes with significantly negative (PMi<−0.5; *RPL30*, *RPL28*, *RPL33A*), significantly positive (PMi>0.5; *YDR367W*) and with less important changes (−0.3<PMi<0.3; *RPL34A*, *RPL33B*) in the PMi, as reported with the tiling arrays. Moreover, we analyzed the changes in the intron/exon signals at different osmotic stress treatment times. As seen in Figure 2A, the results reveal a sharp drop in the RP pre-mRNAs of genes *RPL30*, *RPL28* and *RPL33A* during the first 15 min of osmotic stress, where the minimum value was reached after 10 min, and the corresponding PMis were in agreement with our genomic data. For genes *RPL34A* and *RPL33B*, we also observed a slight drop in the pre-mRNA levels at 5, 10 and 15 min of stress, and we obtained a lower negative PMi at 15 min as compared to the previous RP genes (Fig. 2A). A less pronounced reduction in total mRNA was observed for all these RP genes. Finally, the pre-mRNA levels of *YDR367W* increased during osmotic stress and showed a positive PMi (Fig. 2A), which is in agreement with the tiling data. Thus, the quantitative RT-PCR results reinforce the idea that RP mRNAs have a negative PMi, which strikingly contrasts to the remaining ICGs. Moreover, these results suggest that the drop observed in the RP intron/exon ratio during osmotic stress with the tiling array data is caused by a rapid reduction in the ribosomal protein unspliced transcript levels.

Under osmotic stress conditions, changes in gene expression are controlled mostly by the Hog1 MAP kinase [28]. Recently, we described how the cap-binding protein Cbc1 is involved in the rapid reprogramming of translation under osmotic stress [33]. Additionally, Cbc1 has been described to be required for the proper splicing of some RP genes [37]. To check whether Hog1 and/or Cbc1 play a role in the drop of RP pre-mRNAs under osmotic stress, we calculated the PMi for several RP genes in mutants *hog1* and *cbc1*. In the *hog1* mutant cells, the high negative PMis for *RPL30*, *RPL28, RPL37A* and *RPL33A* were similar to those in the wild-type cells, indicating that the reduction of intron signal in relation to exon signal during osmotic stress is independent of the presence of MAP kinase Hog1 (Fig. 2B). Some differences in the PMi values were obtained for some specific RP genes in both mutants in relation to the wild-type values, such as *RPS13 and RPL33B* in *hog1*, and *RPS13, RPL33A* and *RPL33B* in the *cbc1* mutant. Despite these differences, the remaining RP genes analyzed in the mutants gave similar negative PMi values to those in the wild-type cells. These results indicate that the sharp drops in some RP pre-mRNA levels still occur in mutants *hog1* and *cbc1*, although there are differences between these mutants and the wild-type PMi for specific RP genes. Therefore, these data suggest that deletion of Hog1 or Cbc1 does not suppress all the changes occurring in the RP pre-mRNA relative levels under osmotic stress.

### Decreases in RP Pre-mRNA during Osmotic Stress are not Due to Lower Transcription Rates

**Figure 3:**
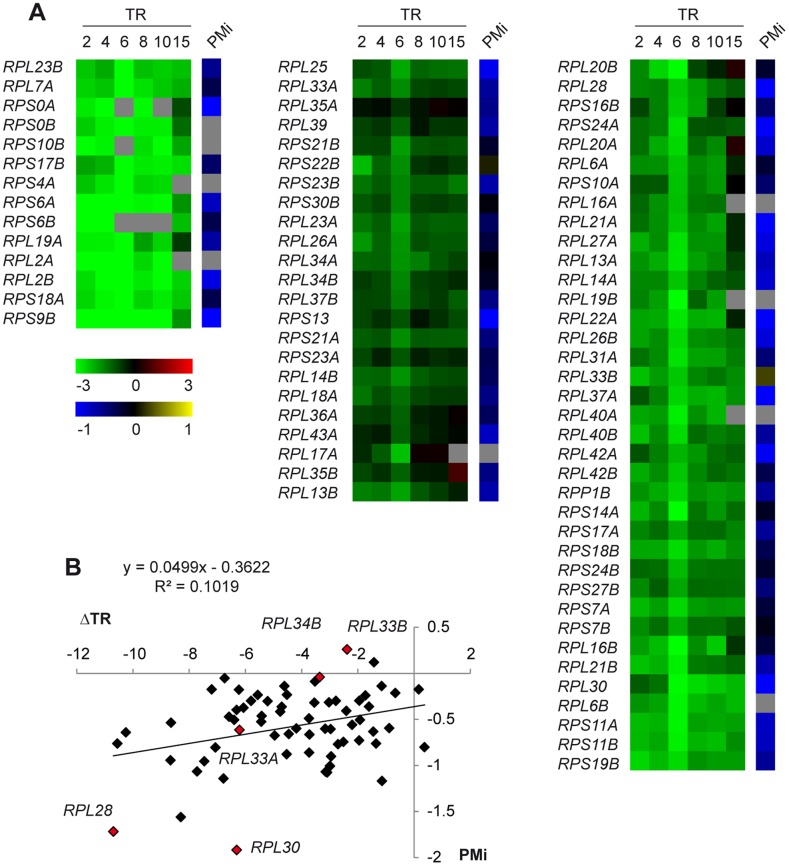
Analysis of the relation between ribosomal protein genes transcription and pre-mRNA processing during osmotic stress. (A) Clustering RP genes according to their transcription rate (TR) profiles. Wild-type cells were grown in YPD until the exponential phase and were then treated with 0.4 M NaCl. The samples taken at 0, 2, 4, 6, 8, 10 and 15 minutes of osmotic shock were processed to measure the TR of all the yeast genes. The data set series for intron-containing RP genes refers to their respective 0 time on a logarithm scale. A relative repression (stress/non-stress ratio, on the log_2_ scale) is shown in green (saturated green indicates a decrease of at least 6-fold) and relative induction is depicted red (saturated red indicates an increase of at least 6-fold). RP genes were ordered and grouped into three distinct subclasses by K-means clustering using Euclidean distance. The PMi for all the RP genes are represented in the left panel using a blue-yellow heat map (saturated blue indicates a PMi of ≤−1, while saturated yellow indicates a PMi of ≥1). Grey squares indicate missing values. (B) Comparison of RP genes ΔTR and their PMi in the cells treated with 0.4 M NaCl for 15 minutes related to non-stressed cells. The regression line, equation and correlation coefficient are shown in the graph. The RP genes that have been analyzed by quantitative RT-PCR are indicated as red diamonds.

Recently, another group analysed the changes in the pre-mRNA and mRNA levels under stress conditions [12]. This group found that RP pre-mRNA levels dropped under osmotic stress conditions, which is in agreement with our data. The authors proposed such decreases might be due to the drop in the rate of new transcription. To study this possibility, we analyzed the relation between the kinetics of the RP gene transcription rate (TR) after treatment with 0.4 M NaCl, obtained by genomic run-on [30], and the PMi values. The RP genes classified into three groups according to TR kinetics (see Materials and methods for a detailed explanation). The TR also lowered in almost all the RP genes, which was more or less severe depending on the group (Fig. 3A). However, the distribution of the PMi values for the RP gene members of the three groups seemed to be random, showing no clear pattern (Fig. 3A). We also analyzed the correlation between the changes in the TR and the PMi values after 15 minutes of the NaCl treatment (Fig. 3B). The correlation was very poor (r^2^ = 0.1019) and a t-score statistic test of the tendency line indicated that the regression slope did not significantly differ from zero. Hence, this analysis indicates that the lowered RP pre-mRNA levels upon osmotic stress were not due to a drop in the RP gene transcription rates.

### Non-sense Mediated Decay (NMD) Reduces RP Pre-mRNA Levels during Osmotic Stress

**Figure 4:**
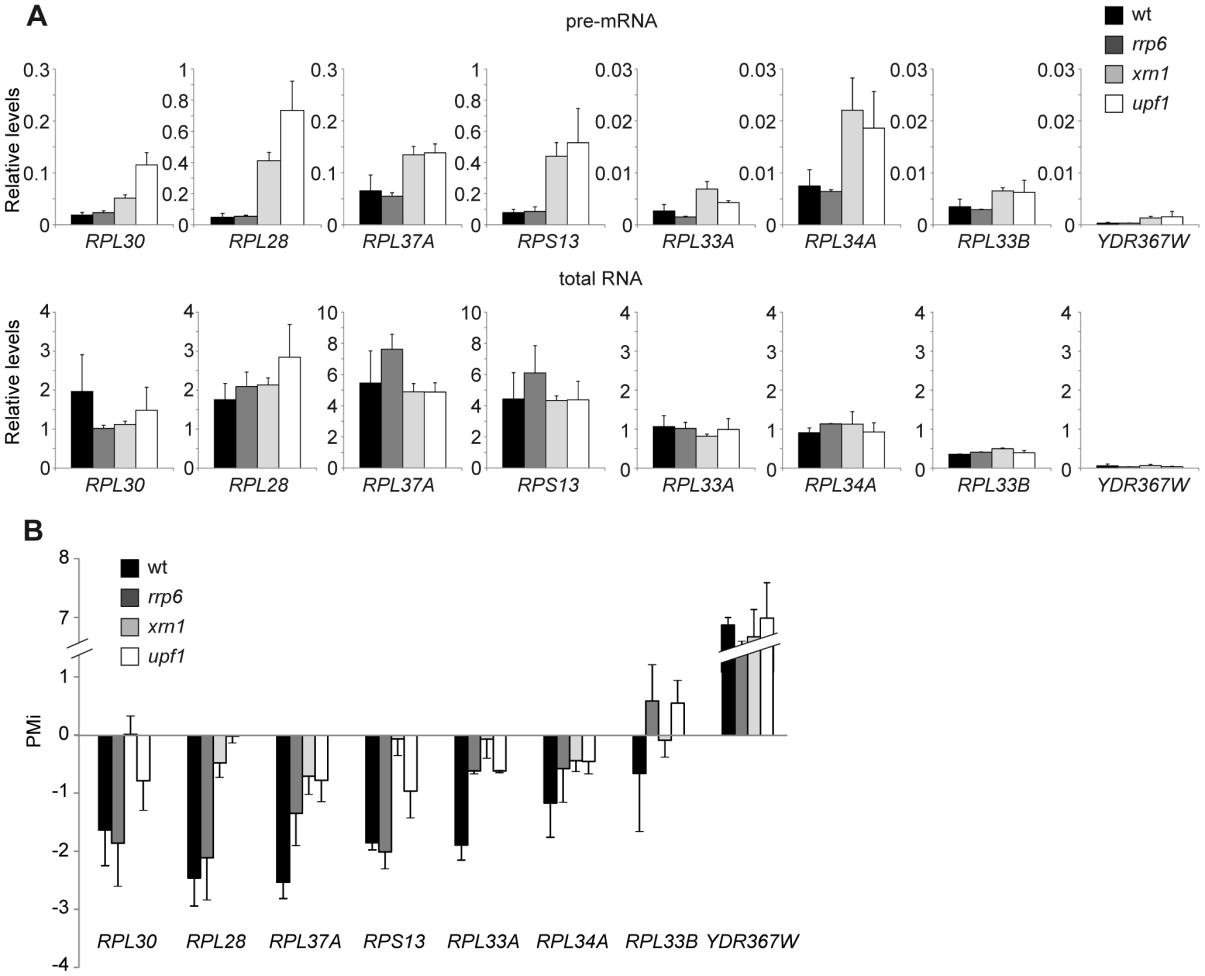
Upf1 and exonuclease Xrn1 contribute to the decrease in ribosomal protein pre-mRNA during osmotic stress. (A) The total and pre-mRNA relative levels are shown for the non-stressed cells of a wild-type strain and strains with deletions in *RRP6*, *XRN1* or *UPF1.* (B) Representation of PMi in the cells treated with 0.4 M NaCl for 15 minutes in relation to untreated cells for the wild-type strain and the same mutant strains as in (A). The intron and exon levels of the transcripts *RPL30*, *RPL28*, *RPL37A*, *RPS13*, *RPL33A*, *RPL34A, RPL33B* and *YDR367W* were examined by quantitative RT-PCR using specific primers to those regions. Data and errors bars represent the average and standard deviation of 3 independent experiments.

The negative PMi for the RP genes may indicate that RP pre-mRNA levels lower by either a greater splicing efficiency or a higher pre-mRNA degradation rate during osmotic stress. To discriminate between these two possibilities, first we wondered whether the RP genes showing a negative PMi were targeted by nuclear and cytoplasmic degradation pathways under normal conditions. Figure 4A shows the pre-mRNA and total mRNA levels in the strains with deletions in *RRP6*, encoding a component of the nuclear exosome [38]; *XRN1*, encoding a cytoplasmic 5′ to 3′ exonuclease [39]; or *UPF1*, encoding a component of the cytoplasmic nonsense-mediated mRNA decay pathway [40]. The results show increased RP pre-mRNA levels for all the RP genes tested in the mutants *xrn1* and *upf1*, but no differences were seen in the *rrp6* mutant in relation to the wild-type levels (Fig. 4A). The involvement of Xrn1 and Upf1 in the regulation of RP pre-mRNAs under non-stress conditions is in agreement with the results previously obtained by Sayani *et al*. [23]. According to their data, *RPL28*, *RPL30* and *RPS13* are targets of Xrn1 and Upf1. However, our results indicate that *RPL37A*, *RPL33A*, *RPL34A* and *RPL33B* are also dependent on both factors; meanwhile, Sayani *et al.* [23] described these four RP genes as Upf1-independent and only the last three as Xrn1-dependent. Next, we calculated the PMi for the mRNA processing mutants to determine the decrease in the pre-mRNA levels during osmotic stress. The negative PMis for all the RP genes tested significantly reduced in mutants *xrn1* and *upf1* (Fig. 4B). However, the mutant *rrp6* only showed an effect on the PMi of some RP genes (Fig. 4B). These results suggest that the drop in the RP pre-mRNA levels is due to a higher RNA degradation rate during osmotic stress since the RP gene PMi values were close to zero in those cells with a mutation in the *XRN1* or *UPF1* decay factors. Moreover, our results indicate that the NMD pathway controls the RP pre-mRNA levels in the wild-type strain under normal conditions and also during response to osmotic stress. Additionally, although the change in the intron/exon ratios during osmotic stress was affected for specific RP genes in mutant *rrp6,* the nuclear degradation of RP pre-mRNAs by Rrp6 does not seem to explain the whole global regulation of RP pre-mRNA levels. A further genomic analysis in mutant *rrp6* would be required to determine the contribution of Rrp6 to the regulation of each specific yeast RP pre-mRNA.

**Figure 5:**
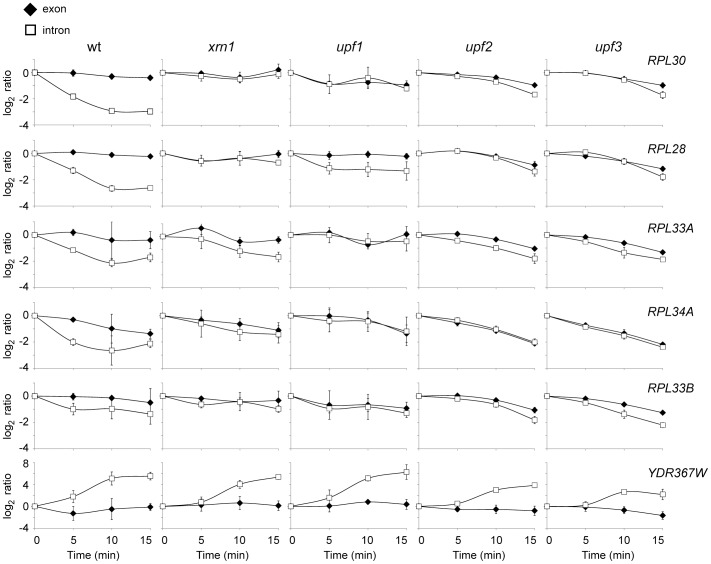
Kinetics of ribosomal protein pre-mRNAs during osmotic stress in cells containing mutations in NMD components. The behaviour of the transcripts *RPL30*, *RPL28*, *RPL33A*, *RPL33B*, *RPL34A* and *YDR367W* after 0, 5, 10 and 15 minutes treatment with 0.4M NaCl were examined in the wild-type, *upf1*, *upf2* and *upf3* and *xrn1* mutants by quantitative RT-PCR, as described in Figure 2A. Data and errors bars represent the average and standard deviation of 3 independent experiments.

To further confirm the role of NMD in the regulation of the RP pre-mRNA levels during osmotic stress, we investigated the kinetics of the intron and exon levels of RP genes and of a control gene in those strains with single mutations in all the core components of the pathway: Upf1, 2 and 3, and also in the NMD-related exonuclease Xrn1. The drop in the pre-mRNA levels of *RPL30*, *RPL28*, *RPL33A*, *RPL34A* and *RPL33B* observed in the wild-type strain after treatment with 0.4 M NaCl was significantly reduced in mutant strains *xrn1*, *upf1*, *upf2* and *upf3* (Fig. 5). However, the increase in the pre-mRNA levels of the *YDR367W* gene was not affected in the NMD mutants (Fig. 5). All together, we conclude that the cytoplasmic NMD pathway is responsible for, or contributes substantially to, the decrease in RP pre-spliced transcripts that occurs in response to osmotic stress.

## Discussion

This study presents the analysis of changes in the pre-mRNA/mRNA ratios during hyperosmotic stress (PMi) for all the intron-containing genes using tiling arrays. Our results clearly reveal that the pre-mRNA relative levels of the RP genes group lower in response to osmotic stress. These results are in accordance with the previous results recently shown by Bergkessel *et al*. [12]. In their work, the authors analyzed the changes in pre-mRNA processing under diverse environmental conditions using splicing-specific microarrays, and they found distinct responses; for example, accumulation of RP pre-mRNAs during amino acid starvation and reduced RP pre-mRNAs under hyperosmotic stress. The authors proposed that the osmotic stress-dependent decrease was the result of a drop in the RP genes transcription rates [12]. However, we found that the lower RP-unspliced relative levels was due to an increase in their degradation rates.

**Figure 6:**
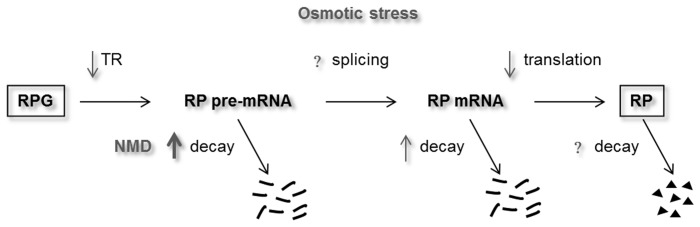
Scheme of the changes in ribosomal protein (RP) gene expression upon osmotic stress. The RP pre-mRNA steady-state depends on both the RP gene transcription rate (TR) by RNA polymerase II and the RP pre-mRNA splicing and degradation rates. The RP mRNA produced by splicing can be used for the synthesis of ribosomal proteins through translation or can be sent to degradation. Osmotic stress provokes a decrease in the RP gene TR and an NMD-dependent increase in the RP pre-mRNA degradation rate. Additionally, osmotic stress increases the RP mRNA degradation rate (lower than the increase in the pre-mRNA degradation rate) and lowers the RP mRNA translation rate. The effect of osmotic stress on RP pre-mRNA splicing and RP decay has not been directly estimated. As a result of these changes, the RP pre-mRNA levels dropped considerably and, to a lesser extent, the RP mRNA level also lowered under osmotic stress. However, RP levels remained constant as a result of the growth inhibition provoked by osmotic stress. See the text for further discussion.

The steady-state of the RP pre-mRNA levels depends on the TR at which RP pre-mRNAs are produced by RNA polymerase II, the splicing rate at which RP-pre-mRNAs are converted into mature RP mRNAs and the rate at which RP pre-mRNAs are degraded (Fig. 6). Our previous study showed that hyperosmotic stress yields a global reduction in the genes TR, including the transcription of RP genes [30]. In the present study we analyzed the TR kinetics during osmotic stress for all RP genes and compared these data with the PMis calculated for all the RP genes. The correlation between the drop in TR and PMi was very poor, indicating that the decrease in RP pre-mRNA levels is not caused mainly by the drop in transcription. Conversely, we found that the mutation of any of the NMD decay pathway components, *UPF1*, *2* and *3* or of the 5′ to 3′ exonuclease *XRN1*, eliminated the decrease in the RP pre-mRNAs during osmotic stress. Therefore, the drop in RP intron-containing transcripts is due to an increased pre-mRNA degradation rate conducted by the NMD pathway (Fig. 6). It has been previously shown [29], [30] that RP mRNAs stability also transiently decreased during osmotic stress. As the disappearance of mature RP mRNA acts to increase the proportion of RP pre-mRNAs, it can be concluded that osmotic stress provokes an increase in both the RP pre-mRNA and mRNA degradation rates, but the increase in the former is so high that it surpasses the effect on mature RP mRNA destabilization (Fig. 6).

In principle, degradation of unspliced transcripts could occur in the nucleus through the nuclear exosome or other nuclear degradation systems [41], [42], [43], or in the cytoplasm through NMD or other alternative cytoplasmic degradation pathways [16], [44], [45]. Our results indicate that the drop of RP pre-mRNAs during osmotic stress is mainly the result of a higher cytoplasmic degradation rate through the NMD surveillance pathway. The increased turnover of RP pre-mRNAs by NMD might be due to an enhanced NMD activity or to enhanced delivery of unspliced RP transcripts to the cytoplasm. This second possibility implies that the degradation rate by NMD is higher than the initial nuclear degradation rate. Increasing arrivals of RP pre-mRNAs at the cytoplasm can be explained, for example, by the failure of spliceosomes to assemble on RP pre-mRNAs under stress conditions since spliceosomes assembly has been reported to prevent the export of mRNA before splicing. Another alternative explanation is that the nuclear degradation by the exosome is inhibited by osmotic stress. In this case, since it has been reported that nuclear exosome and NMD can degrade unspliced transcripts in a sequential and complementary manner [46], a higher fraction of RP unspliced transcripts would be targeted by NMD.

The first possibility outlined above, that is, greater NMD pathway activity during osmotic stress, would explain the drop in RP pre-mRNAs since, as reported here and previously [23], [46], many RP unspliced transcripts are already targets of NMD under non-stress conditions. Similarly in *C. elegans* [47], Paramecia [48] and mammalian cells [24], [25], it has been shown that NMD regulates the pre-mRNA levels of ribosomal and non-ribosomal genes. Interestingly, there is increasing data in yeast and mammals to suggest that the NMD pathway regulates gene expression under stress conditions. For example, NMD prevents the accumulation of unspliced RP transcripts during amino acid starvation, when splicing is inhibited [11], [23]. In mammalian cells under hypoxia, the NMD pathway is regulated to yield the stabilization of stress-responsive mRNAs [25]. Along these lines, the particular regulation of the RP gene expression by NMD under osmotic stress may indicate a variation in NMD activity during this stress. If there is greater NMD activity during osmotic stress as a result of the regulation of splicing efficiency, it should be further studied (Fig. 6). However, the fact that the changes in the RP pre-mRNA/mRNA ratios under osmotic stress (PMi) disappeared in upf mutants argued against a change in RP splicing efficiency during osmotic stress. Moreover, our genomic data do not indicate a clear regulation pattern of the expression levels of the yeast spliceosomal components during osmotic stress (Fig. S1).

One important question to emerge from our results is the biological significance of RP gene regulation by NMD. RP genes are one of the most highly expressed groups in all living organisms; therefore, their expression constitutes a high energy cost for cells. Thus, a fine-tuning regulation of the ribosomal protein gene expression might prove crucial under conditions that compromise survival. The NMD pathway may reduce the levels of RP pre-mRNAs upon osmotic stress to benefit stressed cells in different ways. First, less splicing machinery might be compromised for ribosomal proteins production. Second, the regulation at the pre-mRNA level may allow a less marked reduction in RP gene TRs for the down-regulation of the RP transcript levels during stress, thus making a quicker recovery of the RP gene transcription after adaptation possible. Third, and perhaps more importantly, the reduction of RP pre-mRNAs, together with the destabilization of mature RP transcripts [29], [30], may serve to redirect the translational capacity to the newly synthesized osmo-stress response mRNAs [31], [32], [33]. Thus, large amounts of osmo-protective proteins are quickly obtained [49], [50]. In fact, Lee *et al*. [49] demonstrated that the final goal of reducing RP transcript levels during osmotic stress is not the reduction of RP, because the cellular content of ribosomal proteins remains constant during stress due to transient growth arrest (Fig. 6). These authors estimated, however, that the fraction of translating ribosomes which become available due to transcript reduction accounts for the ribosomes required to translate newly made mRNAs. Thus, a higher degradation rate of unspliced and mature RP transcripts would provide more free ribosomes. One problem with this hypothesis is the low quantitative impact of reducing RP pre-mRNA on competition for available ribosomes, by taking into account that, in any case, the proportion of RP pre-mRNA is very small in relation to the total RP transcripts. Alternatively to a quantitative negative effect, RP pre-mRNAs may possess a specific characteristic which would compromise the stress response. Interestingly, our data suggest that the down-regulation of RP pre-mRNA levels during osmotic stress is Hog1 independent. The MAP kinase has proved essential for transcription reprogramming [30], but it is not required for the global and transient inhibition of translation upon osmotic stress [31], [32], [51]. Together, all these data suggest that upon osmotic shock, the initial clearance of ribosomes from pre-existing RNAs is regulated by a Hog1-independent pathway, which will allow a later and Hog1-dependent association of ribosomes with newly synthesized stress-responsive mRNAs.

It is gradually becoming clear that the presence of introns in most *S. cerevisiae* RP genes, despite the small number of intron-containing genes in yeast [3], serves as an additional and important element for regulating the RP gene expression. To support this, RP intron deletion studies indicate that most introns are required for optimal cell fitness or growth under stress [4]. Moreover, the regulation of ribosomal protein gene expression in *S. pombe* cells under heat-shock is controlled at the pre-mRNA turnover level and requires the presence of introns [43]. Regulation of the *S. cerevisiae* RP gene expression at very different levels in response to osmotic stress, e.g. transcription initiation [30], transcription elongation [7], pre-mRNA stability ([12] and this work), mRNA stability [29], [30], and translation [31], [32] (Fig. 6), exemplifies the need of a multi-level fine-tuning regulation of the RP gene expression in response to environmental changes and the role of introns in this tight regulation.

## Supporting Information

Figure S1 **Analysis of the expression of spliceosome genes during osmotic stress.** (A) Average of mRNA levels for the spliceosomal complex genes (53 out of 58 described) during osmotic stress. Wild-type cells were grown in YPD until exponential phase and then treated with 0.4 M NaCl. Samples taken at 0, 2, 4, 6, 8, 10 and 15 minutes of osmotic shock were processed to measure mRNA levels of all yeast genes. Bars represent Standard errors. The average of the spliceosomal component genes shows a flat profile indicating no general regulation of this gene set. (B) Clustering of spliceosomal complex genes according to their mRNA level profiles. Data set series for spliceosomal complex genes (same data set used in A) refer to their respective time 0 on a logarithm scale. Relative repression (stress/non-stress ratio, in log_2_ scale) is shown in green (saturated green indicates a decrease of at least a 4-fold) and relative induction is depicted red (saturated red indicates an increase of at least a 4-fold). Spliceosomal complex genes were ordered and grouped into subclasses by K-means clustering using the Euclidean distance. The change in individual mRNA levels with regard time zero is low and there are multiple small clusters that show internal division of the gene set.
(TIF)

Table S1 Primers used in this work.
(DOCX)

Table S2 PM index list calculated using the exons and introns intensity signals after 15 min of osmotic stress (0.4 M NaCl) relative to non-stress conditions obtained from tiling arrays.
(DOCX)
