# Supplementary figures and images for: Nonsense-Mediated mRNA Decay Controls the Changes in Yeast Ribosomal Protein Pre-mRNAs Levels upon Osmotic Stress
Source: PLoS One. 2013 Apr 19;8(4):e61240. doi: 10.1371/journal.pone.0061240 (PMC3631235; doi:10.1371/journal.pone.0061240)

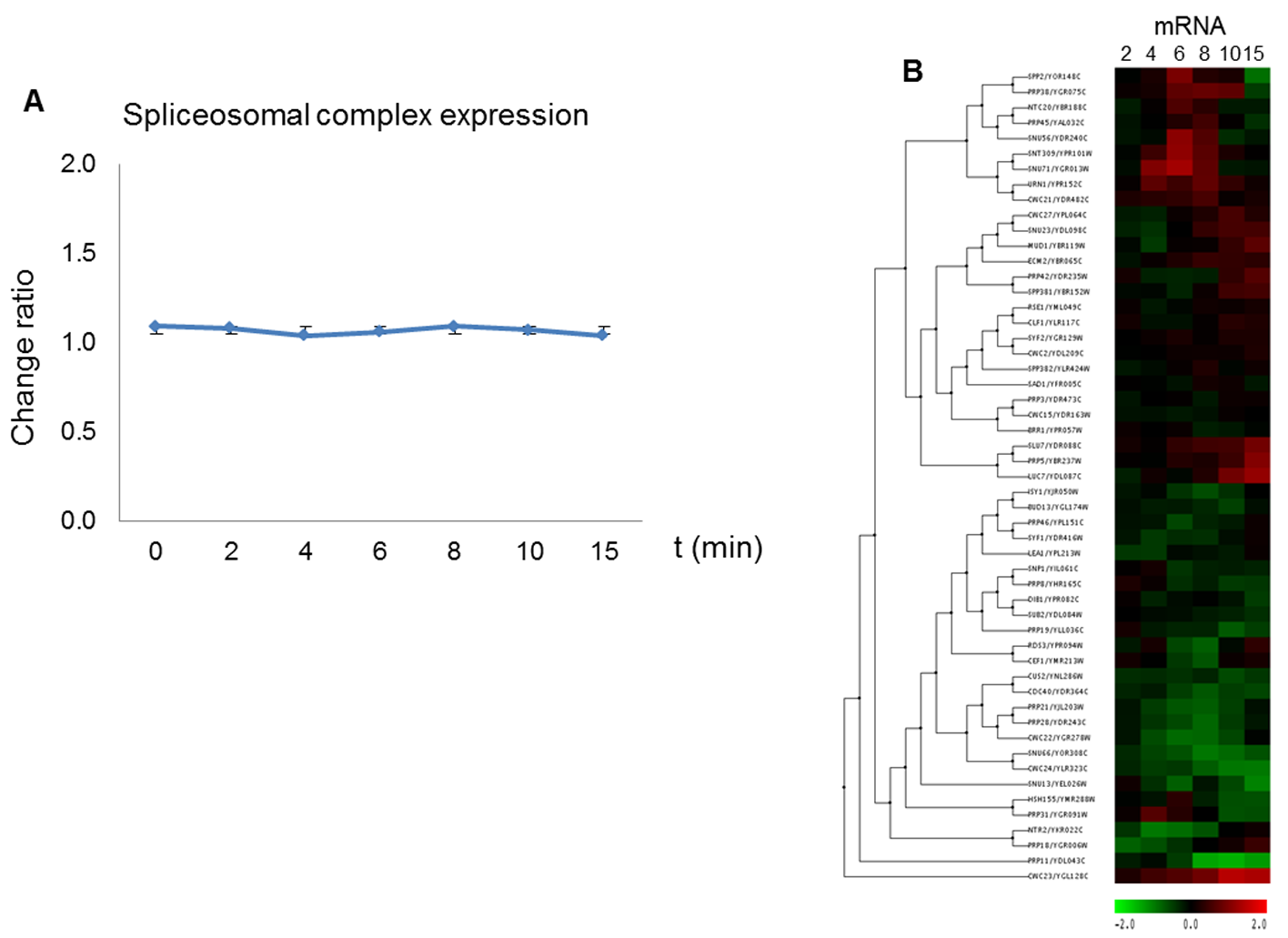

Supplement: Figure S1 — Analysis of the expression of spliceosome genes during osmotic stress. (A) Average of mRNA levels for the spliceosomal complex genes (53 out of 58 described) during osmotic stress. Wild-type cells were grown in YPD until exponential phase and then treated with 0.4 M NaCl. Samples taken at 0, 2, 4, 6, 8, 10 and 15 minutes of osmotic shock were processed to measure mRNA levels of all yeast genes. Bars represent Standard errors. The average of the spliceosomal component genes shows a flat profile indicating no general regulation of this gene set. (B) Clustering of spliceosomal complex genes according to their mRNA level profiles. Data set series for spliceosomal complex genes (same data set used in A) refer to their respective time 0 on a logarithm scale. Relative repression (stress/non-stress ratio, in log2 scale) is shown in green (saturated green indicates a decrease of at least a 4-fold) and relative induction is depicted red (saturated red indicates an increase of at least a 4-fold). Spliceosomal complex genes were ordered and grouped into subclasses by K-means clustering using the Euclidean distance. The change in individual mRNA levels with regard time zero is low and there are multiple small clusters that show internal division of the gene set. (TIF) [file pone.0061240.s001.tif]
