# Supplementary material for: Nonsense-Mediated mRNA Decay Controls the Changes in Yeast Ribosomal Protein Pre-mRNAs Levels upon Osmotic Stress
Source: PLoS One. 2013 Apr 19;8(4):e61240. doi: 10.1371/journal.pone.0061240 (PMC3631235; doi:10.1371/journal.pone.0061240)
Supplement: Table S1 — Primers used in this work. (DOCX) [file pone.0061240.s002.docx]

| **Primer** | **Sequence** |
| --- | --- |
| *ACT1*_exon-F | TCGTTCCAATTTACGCTGGTT |
| *ACT1*_exon-R | CGGCCAAATCGATTCTCAA |
| *RPL28*_exon-F | AAGCAACAAGCTCATTTCTGGAA |
| *RPL28*_exon-R | TGGGATCAATGTCCACAATTTG |
| *RPL28*_intron-F | CACTGTTGAGACGGCTTATTTGA |
| *RPL28*_intron-R | TTGCTCAGTTTGCGATGGAA |
| *RPL30*_exon-F | ACTTCCAAGGTGGTAACAACGAA |
| *RPL30*_exon-R | AAATAGAGACAACACCGACTCTGAATAA |
| *RPL30*_intron-F | TCCTGCTCTTGTGTTTGAGAGAGT |
| *RPL30*_intron-R | GCATATCTCACTTTTATTTCACAGTCATG |
| *RPL33A*_exon-F | CCAAAGATCCAAGAGAGTCAACAA |
| *RPL33A*_exon-R | TGGAGTAGCGACACCTTCGA |
| *RPL33A*_intron-F | CGTGGTGACTAAAATGTGCAAGA |
| *RPL33A*_intron-R | CTTTAGCTGTCTGGTGTTAAATCTGAGT |
| *RPL33B*_exon-F | TGGTGTCGTTAGAGCTACCTTTAGAA |
| *RPL33B*_exon-R | TTCTGACAGAGGCACCGAAA |
| *RPL33B*_intron-F | TGCGTCCCTTTCCATCATG |
| *RPL33B*_intron-R | ATGAGCACGTTGCAGAGTGAA |
| *RPL34A*_exon-F | TTTTGCGTGCCCAACATG |
| *RPL34A*_exon-R | CGCTACCACAGTCACCACACTT |
| *RPL34A*_intron-F | CGCGAAATATTGTGTCCAGAAA |
| *RPL34A*_intron-R | CAGGGTTAAAAGTGGCTGCTCTT |
| *RPL37A*_exon-F | ACAGATGTGGTCGTCGTTCT |
| *RPL37A*_exon-R | CAGCTGGATAACCACAGGAG |
| *RPL37A*_intron-F | GCAAGACTGGACGATACGAG |
| *RPL37A*_intron-R | CGCATGTCTTTCGTTCACTA |
| *RPS31*_exon-F | CAGCTTGGTTCAAGTTGTCC |
| *RPS31*_exon-R | AAACCCTTTCTCGCGTACTT |
| *RPS31*_intron-F | TTGAAGAATCACCCGGAATA |
| *RPS31*_intron-R | CATATTTCCTCGTTCAGACCA |
| *YDR367W*_exon-F | ATGGTTCACCTTGGAAGACA |
| *YDR367W*_exon-R | CAGCTTACCTGTCGATATTGG |
| *YDR367W*_intron-F | TCCGGCAAACGCTATAAAT |
| *YDR367W*_intron-R | GTAAAATTGTGCTTTGCTTCG |

**Table S1.** Primers used in this work.
