# Supplementary material for: Nonsense-Mediated mRNA Decay Controls the Changes in Yeast Ribosomal Protein Pre-mRNAs Levels upon Osmotic Stress
Source: PLoS One. 2013 Apr 19;8(4):e61240. doi: 10.1371/journal.pone.0061240 (PMC3631235; doi:10.1371/journal.pone.0061240)
Supplement: Table S2 — PM index list calculated using the exons and introns intensity signals after 15 min of osmotic stress (0.4 M NaCl) relative to non-stress conditions obtained from tiling arrays. (DOCX) [file pone.0061240.s003.docx]

**Table S2.** PM index list calculated using the exons and introns intensity signals after 15 min of osmotic stress (0.4 M NaCl) relative to non-stress conditions obtained from tiling arrays.

| **Gen** | **PMi** | **Gen** | **PMi** | **Gen** | **PMi** |
| --- | --- | --- | --- | --- | --- |
| *RPL30* | -1.92 | *RPL37B* | -0.53 | *RPL26A* | -0.24 |
| *RPL28* | -1.72 | *MOB1* | -0.51 | *TUB1* | -0.24 |
| *RPS13* | -1.56 | *RPL18A* | -0.51 | *RPL7A* | -0.23 |
| *RPS9B* | -1.17 | *RPS21A* | -0.49 | *RPS16B* | -0.23 |
| *RPL37A* | -1.14 | *YBR062C* | -0.47 | *HMRA1* | -0.23 |
| *SAE3* | -1.12 | *RPS27B* | -0.47 | *RPS19A* | -0.22 |
| *RPL21A* | -1.08 | *RPL31A* | -0.46 | *RPS6A* | -0.21 |
| *RPL22A* | -1.07 | *RPS10B* | -0.44 | *RPS30A* | -0.21 |
| *RPS24A* | -1.06 | *YKR005C* | -0.44 | *MRK1* | -0.21 |
| *RPS0B* | -1.01 | *YDR381C-A* | -0.42 | *OSW2* | -0.20 |
| *RPL42A* | -0.96 | *AMA1* | -0.42 | *YPR063C* | -0.19 |
| *RPL25* | -0.94 | *RPS10A* | -0.42 | *MND1* | -0.19 |
| *RPL2B* | -0.90 | *RPL16B* | -0.41 | *APE2* | -0.19 |
| *RPL27A* | -0.88 | *YLR202C* | -0.41 | *RPS24B* | -0.18 |
| *RPL26B* | -0.86 | *BOS1* | -0.40 | *DCN1* | -0.18 |
| *RPL14A* | -0.81 | *RPS17B* | -0.40 | *RPL20B* | -0.18 |
| *RPL20A* | -0.80 | *RPS4B* | -0.39 | *RPS21B* | -0.17 |
| *RPS11A* | -0.77 | *IST1* | -0.39 | *PCH2* | -0.17 |
| *RPL43A* | -0.76 | *DID4* | -0.39 | *IWR1* | -0.16 |
| *RPL6A* | -0.76 | *RPS0A* | -0.38 | *YBL059W* | -0.16 |
| *RPS11B* | -0.75 | *RPS16A* | -0.38 | *REC102* | -0.15 |
| *RPL13A* | -0.73 | *RPL23A* | -0.37 | *SMD2* | -0.15 |
| *ARP2* | -0.72 | *COF1* | -0.37 | *SRB2* | -0.15 |
| *RPL13B* | -0.67 | *PMI40* | -0.37 | *RPL36B* | -0.14 |
| *RPS23B* | -0.66 | *RPS6B* | -0.37 | *RPL34B* | -0.14 |
| *RPL21B* | -0.66 | *RPL14B* | -0.36 | *MOB2* | -0.14 |
| *RPL39* | -0.64 | *RPL36A* | -0.36 | *RPS14A* | -0.14 |
| *RPL19A* | -0.63 | *OST5* | -0.36 | *REC107* | -0.13 |
| *RPL43B* | -0.63 | *RIM1* | -0.35 | *SPO22* | -0.13 |
| *RPL2A* | -0.62 | *RPL16A* | -0.34 | *ERV41* | -0.13 |
| *RPL33A* | -0.61 | *YJR112W-A* | -0.34 | *RPL18B* | -0.13 |
| *RPL17A* | -0.61 | *KIN28* | -0.34 | *UBC13* | -0.12 |
| *RPL40B* | -0.61 | *REC114* | -0.34 | *UBC4* | -0.12 |
| *RPS14B* | -0.60 | *RPS18B* | -0.32 | *APS3* | -0.11 |
| *RPS17A* | -0.60 | *RPS23A* | -0.32 | *MCM21* | -0.10 |
| *RPP1B* | -0.60 | *RPS7A* | -0.30 | *DMC1* | -0.09 |
| *RPS19B* | -0.59 | *RPL42B* | -0.30 | *HNT2* | -0.09 |
| *RPL40A* | -0.59 | *RPL6B* | -0.30 | *RPS7B* | -0.09 |
| *RPL19B* | -0.59 | *RPS18A* | -0.30 | *NMD2* | -0.08 |
| *BIG1* | -0.56 | *ERV1* | -0.27 | *YML133C* | -0.08 |
| *RPL23B* | -0.56 | *YER093C-A* | -0.26 | *HFM1* | -0.08 |
| *RPL35A* | -0.54 | *YPR170W-B* | -0.25 | *ARP9* | -0.07 |
| *TFC3* | -0.54 | *PTC7* | -0.24 | *HPC2* | -0.07 |

| **Gen** | **PMi** | **Gen** | **PMi** | **Gen** | **PMi** |
| --- | --- | --- | --- | --- | --- |
| *ACT1* | -0.07 | *YEL076C-A* | 0.03 | *TMA20* | 0.17 |
| *BET4* | -0.06 | *VMA10* | 0.03 | *RPL17B* | 0.18 |
| *YBL059C-A* | -0.06 | *YPR153W* | 0.04 | *YBR090C* | 0.19 |
| *UBC12* | -0.06 | *TEF4* | 0.04 | *YIP3* | 0.20 |
| *VPS75* | -0.06 | *YHL050C* | 0.05 | *SAR1* | 0.20 |
| *RPS30B* | -0.05 | *BUD25* | 0.05 | *SFT1* | 0.21 |
| *RPS4A* | -0.05 | *NSP1* | 0.06 | *SPT14* | 0.21 |
| *PRE3* | -0.04 | *YPR010C-A* | 0.06 | *SUS1* | 0.21 |
| *YLL067C* | -0.04 | *YBR219C* | 0.07 | *SRC1* | 0.23 |
| *RPL34A* | -0.04 | *YSF3* | 0.07 | *DTD1* | 0.23 |
| *GCR1* | -0.04 | *PHO85* | 0.07 | *POP8* | 0.23 |
| *PBA1* | -0.04 | *YCL002C* | 0.08 | *DYN2* | 0.24 |
| *GLC7* | -0.03 | *LSM2* | 0.08 | *RPL33B* | 0.26 |
| *YRF1-7* | -0.03 | *YLR211C* | 0.08 | *YLR445W* | 0.28 |
| *SEC14* | -0.02 | *YJR079W* | 0.09 | *PSP2* | 0.30 |
| *SEC27* | -0.02 | *YOS1* | 0.09 | *YLR426W* | 0.30 |
| *CNB1* | -0.01 | *SCS22* | 0.09 | *RUB1* | 0.31 |
| *YJL225C* | -0.01 | *YML6* | 0.09 | *CGI121* | 0.31 |
| *VMA9* | -0.01 | *EPT1* | 0.09 | *TUB3* | 0.34 |
| *YLL066C* | -0.01 | *HOP2* | 0.09 | *TAD3* | 0.40 |
| *YLR464W* | -0.01 | *YPR202W* | 0.09 | *RFA2* | 0.41 |
| *YBL111C* | -0.01 | *ASC1* | 0.09 | *YRA1* | 0.42 |
| *EFB1* | -0.01 | *YBR255C-A* | 0.10 | *TAN1* | 0.45 |
| *YHR218W* | -0.01 | *HRB1* | 0.10 | *NCE101* | 0.51 |
| *ECM9* | -0.01 | *YRF1-3* | 0.10 | *MEI4* | 0.51 |
| *BET1* | 0.00 | *HAC1* | 0.10 | *CIN2* | 0.56 |
| *YRF1-6* | 0.00 | *GIM5* | 0.10 | *NCB2* | 0.59 |
| *PFY1* | 0.01 | *GPI15* | 0.12 | *PCC1* | 0.62 |
| *CPT1* | 0.01 | *YOR318C* | 0.12 | *ERD2* | 0.75 |
| *YIL177C* | 0.01 | *RPS22B* | 0.12 | *CBC1* | 0.75 |
| *YGR001C* | 0.01 | *YIL156W-B* | 0.12 | *UBC9* | 0.87 |
| *GOT1* | 0.01 | *GIM4* | 0.13 | *LSM7* | 0.87 |
| *YNL050C* | 0.01 | *TAF14* | 0.13 | *YDR367W* | 1.11 |
| *YCL012C* | 0.02 | *MMS2* | 0.15 | *YDL012C* | 1.25 |
| *ECM33* | 0.02 | *MRPL44* | 0.16 |  |  |
